# Evaluating the effect of Roxadustat on ventricular repolarization in patients undergoing peritoneal dialysis

**DOI:** 10.1186/s40001-023-01368-0

**Published:** 2023-10-11

**Authors:** Yangyang Zhang, Liang Zhang, Pengcheng Ge, Ruyi Xu, Zhen Ye

**Affiliations:** 1 Department of Cardiology, Suqian First Hospital, Suqian, Jiangsu China; 2 Department of Pharmacy, Suqian First Hospital, 120 Suzhi Road, Sucheng District, Suqian, 223800 Jiangsu China; 3 Department of Nephrology, Suqian First Hospital, Suqian, Jiangsu China

**Keywords:** Roxadustat, Ventricular repolarization, Peritoneal dialysis, Electrocardiogram, Coronary atherosclerosis

## Abstract

**Background:**

Roxadustat is a novel oral medication used to treat anemia in CKD patients. Several studies have shown that Roxadustat can alleviate anemia in CKD patients by increasing hemoglobin levels and regulating iron metabolism. We aimed to evaluate the effect of Roxadustat on ventricular repolarization in PD patients. This study may provide a new integrated approach to the assessment and treatment of CKD.

**Methods:**

The present prospective cohort study enrolled 65 CKD patients who were treated with Roxadustat and 31 CKD patients who received conventional therapy between January 2021 and June 2022. All patients were examined for ECG in the absence of clinical symptoms and compared the ECG indicators. Demographic and clinical data of all patients were collected. All data used SPSS 18.0 for statistical analyses.

**Results:**

The T peak-to-end (Tpe) of PD patients in the Roxadustat group was remarkably slower than that of patients in the conventional group. Additionally, the Tpe/QT ratio in the conventional group was significantly elevated than that in the Roxadustat group. The results of logistic regression analysis showed that Tpe (95%CI 1.191 ~ 2.141, *P* = 0.002) and Roxadustat treatment (95%CI 1.357 ~ 42.121, *P* = 0.021) were the risk factors of PD patients with high Tp-e/QT ratio.

**Conclusion:**

In summary, we found that Roxadustat could improve ventricular repolarization in peritoneal dialysis patients, which indicated a potential cardiovascular protective effect of Roxadustat. This study might provide a new integrated approach to the assessment and treatment of CKD.

## Introduction

Cardiovascular disease (CVD), including heart failure, coronary artery disease, and arrhythmias, among others, is a leading cause of morbidity and mortality worldwide [1]. It is estimated that over 17 million people die each year due to CVD [2]. Sudden cardiac death (SCD) refers to sudden death caused by cardiac reasons. The incidence of SCD is significantly higher in dialysis patients. Dialysis patients are often accompanied by hypertension, diabetes, anemia, and electrolyte disorders, and may also suffer from left ventricular hypertrophy, cardiac enlargement, and coronary artery disease, all of which are risk factors for SCD [3]. Additionally, impaired kidney function in dialysis patients can lead to the accumulation of metabolic waste products in the body, which may hurt heart function and increase the risk of SCD [4–6]. Therefore, it is important to closely monitor cardiovascular disease-related risk factors in dialysis patients and intervene promptly to reduce the risk of SCD.

Dialysis patients may exhibit changes in electrocardiogram (ECG) parameters, including heart rate, rhythm, ST segment, and QT interval [7]. For example, chronic kidney disease (CKD) patients may show bradycardia, arrhythmia, and prolonged QT interval [8]. Through ECG monitoring of dialysis patients, doctors can detect and treat cardiovascular diseases earlier, thus reducing the risk of SCD in patients. Reportedly, compared to the use of Tp-e or QT interval alone, Tp-e/QT is considered a more sensitive indicator [9]. Other studies have confirmed an increase in the Tp-e/QT ratio after peritoneal dialysis (PD) [8, 10]. Roxadustat is a novel oral medication used to treat anemia in CKD patients [11]. Several studies have shown that Roxadustat can alleviate anemia in CKD patients by increasing hemoglobin levels and regulating iron metabolism [12–14]. In a recent study, it was found that Roxadustat promotes angiogenesis through activation of the HIF-1a/VEGF/VEGFR2 pathway and accelerates wound healing [15]. However, there is no research reported on whether Roxadustat can stabilize myocardial cells and improve cardiac repolarization.

In this prospective observational cohort research, we aimed to evaluate the effect of Roxadustat on ventricular repolarization in PD patients. This study may provide a new integrated approach to the assessment and treatment of CKD.

## Material and methods

### Subjects

The present prospective cohort study enrolled 65 CKD patients (Roxadustat group) who were treated with Roxadustat between January 2021 and June 2022. We also enrolled 31 CKD patients who were only treated with conventional therapy (conventional group). All study subjects were treated with PD at our institution. The criteria for inclusion included: (1) patients received regular PD therapy ≥ 3 months; (2) Aged 18–80 years; (3) clinically stable for at least 1 month before evaluation. The exclusion criteria included: (1) recent combination of other active bleeding disorders; (2) patients with seriously severe liver, infection, renal, cardiovascular dysfunctions and malignancy; (3) patients with chronic lung disease, atrial fibrillation, bundle branch block, permanent pacemakers, existing implantable cardioverter-defibrillators (ICDs), and those receiving antiarrhythmic medications. Patients in the Roxadustat group oral Roxadustat capsules 3 times a week at an initial dose of 100mg/dose (< 60kg) or 120mg/dose (≥ 60kg) (FibroGen, State Pharmacopoeia H20180024). All patients signed written informed consent. The present study was approved by the Ethical Committee of our Hospital.

Formula (Z_1-α/2_ *$\sqrt{p*(1-p)}$)^2^/δ^2^ was used to calculate the sample size. *Z*_1-α_/2 = 1.96, *δ* = 0.1, in this study, P is expected to be 90%. Therefore, the minimum sample size for both groups is 35 people.

### Electrocardiographic measurement

All patients underwent an electrocardiogram (ECG) and recorded ECG-related indices after PD in the 12th week of treatment. The subjects were recorded on a 12-lead synchronized ECG using a Marquette MAC1200 ECG machine, set at 25 mm/s and 10.0 mm/mV, by a cardiology nurse specialist, and all patients were examined in the absence of clinical symptoms. RR intervals, QT intervals, heart rate, and Tpeak–Tend (Tp–Te) intervals were measured independently and manually by two trained investigators for three consecutive cardiac cycles in leads with a smooth, clear baseline and no contractions, and averaged. If there is any ambiguity about the measurement, ask the clinical cardiologist and determine the final measurement. Tp-e is taken as the value between the peak of the T wave and the end of the T wave. Corrected Tp-e (cTP-e) is obtained by the Bazett formula, cTp-e = Tp-e/√RR. The QT value is taken from the beginning of the QRS wave to the end of the T wave, cQT is obtained by the Bazett formula cQT = QT/√RR. QRS duration, heart rate, and PR interval are also obtained from the ECG.

### Data collection and scale scoring

Demographic and clinical data of all patients were collected, including age, BMI, sex, comorbidity, etc. Routing whole blood test was performed using an automatic biochemical analyzer (Hitachi 7600, Hitachi Corporation, Japan), and the levels of total cholesterol (TC), triglyceride (TG), high-density leptin cholesterol (HDLC), low-density leptin cholesterol (LDLC), hemoglobin (Hb), albumin (ALB), serum creatinine (sCr), fasting plasma glucose (FPG), serum potassium (K), sodium (Na), calcium (Ca), P, phosphate C-reactive protein (CRP), iron, ferritin, BNP and glycated Hb were recorded.

### Statistical analysis

Continuous data were reported as mean ± SD or median (range), depending on their distribution, which was assessed using the Kolmogorov–Smirnov test. Non-normally distributed data were presented as median (range). The Mann–Whitney test was used to compare two groups for non-normally distributed data, while Student’s t-test was used for normally distributed data. Rates were analyzed using Chi-squared analysis. Spearman’s rank correlation was used for correlation analysis between clinical outcomes and ECG indicators. Logistic regression was performed to identify risk factors associated with high Tp-e/QT ratio in patients. A significant difference was defined as *P* < 0.05. All statistical analyses were conducted using SPSS 18.0.

## Results

### Patient selection and baseline characteristics

The present prospective cohort study enrolled 65 CKD patients (Roxadustat group) who were treated with Roxadustat and 31 CKD patients who were only treated with conventional therapy (conventional group) during January 2021 and June 2022. Comparing the demographic and clinical data of the two groups, we found that there were no significant differences between two groups in age, sex, BMI, history of disease and levels of serum biomarkers (Table 1). The age of the Roxadustat group was 47.13 ± 12.29, and BMI was 24.11 ± 3.56. The age of the conventional group was 49.65 ± 12.38, and the BMI was 23.04 ± 2.92. In Roxadustat group, the proportion of hypertension was 96.9%, the proportion of coronary heart disease was 7.6%, the proportion of diabetes was 16.9%. In conventional group, the proportion of hypertension was 96.9%, the proportion of coronary heart disease was 9.6%, and the proportion of diabetes was 22.5%.

**Table 1:** Basic characteristics of all patients

Variable	Roxadustat group, *n* = 65	Conventional group, *n* = 31	*p*
Age, y	47.13 ± 12.29	49.65 ± 12.38	0.351
Sex, male (%)	40 (61.5%)	21 (67.7%)	0.555
BMI	24.11 ± 3.56	23.04 ± 2.92	0.148
History of disease			
Hypertension, *n* (%)	63 (96.9%)	30 (96.9%)	0.969
Coronary heart disease, *n* (%)	5 (7.6%)	3 (9.6%)	0.687
Diabetes, *n* (%)	11 (16.9%)	7 (22.5%)	0.507
TC (mmol/L)	3.83 ± 0.81	4.02 ± 0.88	0.279
TG (mmol/L)	1.70 ± 1.12	1.57 ± 0.63	0.559
LDLC (mmol/L)	2.55 ± 0.62	2.62 ± 0.65	0.585
HDLC (mmol/L)	0.97 ± 0.25	1.00 ± 0.23	0.613
FPG (mmol/L)	5.45 ± 2.18	5.71 ± 2.61	0.606
Hb (g/l)	100.22 ± 20.08	101.84 ± 17.59	0.701
ALB (g/l)	31.85 ± 9.81	34.87 ± 5.18	0.111
sCr (mmol/L)	1035.34 ± 302.13	997.32 ± 302.55	0.566
K (mmol/L)	4.16 ± 0.79	4.22 ± 0.72	0.700
Na (mmol/L)	139.13 ± 3.81	138.85 ± 3.14	0.725
Ca (mmol/L)	2.18 ± 0.17	2.17 ± 0.22	0.812
P (mmol/L)	1.72 (0.99 ~ 3.20)	1.82 (0.72 ~ 2.64)	0.888
Iron (ng/ml)	8.90 (3.60 ~ 18.20)	8.50 (4.40 ~ 74.40)	0.251
Ferritin (ng/ml)	172.30 (11.96 ~ 1437.00)	212.23 (56.72 ~ 1206.00)	0.223
glycated Hb (mmol/L)	5.70 (4.20 ~ 9.30)	5.60 (4.50 ~ 8.60)	0.591
BNP (mg/dL)	166.00 (1.75 ~ 5000)	103.00 (9.00 ~ 5000)	0.094
CRP (mg/dL)	5.00 (1.00 ~ 79.50)	5.00 (0.01 ~ 96.00)	0.431

### Comparisons of ECG indicators between two groups

Further, all patients were examined for ECG in the absence of clinical symptoms and compared the ECG indicators. It was found that the Tpe of PD patients in the Roxadustat group was remarkably slower than that of patients in the conventional group (*p* < 0.05, Table 2). Additionally, the Tpe/QT ratio in the conventional group was significantly elevated than that in the Roxadustat group (*p* < 0.05). No other significant difference between the two groups.

**Table 2:** Comparisons of ECG indicators between two groups

Variable	Roxadustat group, *n* = 65	conventional group, *n* = 31	*p*
Heart rate	74 (51 ~ 96)	71 (59 ~ 103)	0.498
PR (ms)	157.23 ± 21.477	158.77 ± 25.82	0.761
QRS (ms)	86.00 (70.00 ~ 112.00)	84.00 (30.00 ~ 112.00)	0.941
QT (ms)	412.00 (340.00 ~ 530.00)	414.00 (340.00 ~ 592.00)	0.549
QTc (ms)	462.00 (407.00 ~ 554.00)	459.00 (414.00 ~ 648.00)	0.925
Tpe (ms)	84.00 (62.00 ~ 126.00)	92.00 (66.00 ~ 202.00)	0.011
Tpe/QT	0.21 (0.12 ~ 0.31)	0.23 (0.16 ~ 0.34)	0.001

### The relevance between clinical outcomes and ECG indicators

We subsequently used Spearman rank correlation analysis to examine the relationship between clinical outcomes and ECG indicators in CKD patients. As shown in Table 3, we found that QT is negatively correlated with heart rate (*p* < 0.001), FPG (*p* = 0.004), and Ca (*p* = 0.034), while it is positively correlated with PR (*p* = 0.034). Tpe is positively correlated with ALB (*p* = 0.043). Additionally, Tpe/QT is positively correlated with heart rate (*p* = 0.006) and ALB (*p* = 0.020).

**Table 3:** Correlation between clinical outcomes and ECG indicators in CKD patients

Variables	QT		Tpe		Tpe/QT	
	Spearman’s correlation	*P*	Spearman’s correlation	*P*	Spearman’s correlation	*P*
Age	0.168	0.102	0.024	0.815	−0.094	0.360
BMI	0.093	0.368	−0.042	0.684	−0.102	0.321
Heart rate	−0.625	< 0.001	−0.060	0.562	0.280	0.006
PR	0.217	0.034	−0.055	0.598	−0.188	0.066
Hb	−0.139	0.178	−0.053	0.609	0.608	0.508
ALB	−0.079	0.447	0.207	0.043	0.237	0.020
sCr	−0.103	0.318	0.032	0.760	0.061	0.556
TC	−0.006	0.955	−0.165	0.108	−0.113	0.271
TG	−0.069	0.503	−0.069	0.507	−0.008	0.940
HDLC	0.070	0.497	0.083	0.421	0.035	0.733
LDLC	−0.098	0.342	−0.163	0.112	−0.069	0.507
FPG	−0.290	0.004	−0.129	0.211	0.009	0.931
K	−0.037	0.719	0.004	0.901	−0.013	0.901
Na	0.101	0.326	0.079	0.446	0.017	0.870
Ca	−0.216	0.034	0.032	0.756	0.166	0.106
P	0.129	0.212	−0.004	0.968	−0.051	0.624
Glycated Hb	−0.002	0.981	−0.033	0.747	−0.041	0.694
Ferritin	−0.037	0.718	0.048	0.642	0.035	0.735
Iron	0.037	0.720	0.086	0.404	0.033	0.750
BNP	0.145	0.159	0.043	0.680	−0.039	0.707
CRP	0.113	0.274	−0.114	0.269	−0.180	0.078

### Logistic regression for risk factors of patients with high Tp-e/QT ratio

As a prolonged Tp-e/QT segment was defined as > 0.25 [16], we divided all PD patients into Tp-e/QT ratio high group (≥ 0.25) and Tp-e/QT ratio low group (< 0.25) and finally used logistic regression to analyze the risk factors of patients with high Tp-e/QT ratio. For logistic regression, we used two models using the entry method, including model 1 clinical data (Age, Sex, BMI, history of disease, serum biomarkers and Roxadustat treatment), model 2 ECG indicators (heart rate, PR, QRS, Qtc, Tpe and Tpe/QT). The results showed that Tpe (95% CI 1.191 ~ 2.141, *P* = 0.002) and Roxadustat treatment (95% CI 1.357 ~ 42.121, *P* = 0.021) were the risk factors of PD patients with high Tp-e/QT ratio (Table 4).

**Table 4:** Logistic regression for risk factors of patients with high Tp-e/QT ratio

Variables	Wald	Odds ratio	95% CI	*P*
Age	2.913	0.915	0.826 ~ 1.013	0.088
BMI	0.020	0.977	0.706 ~ 1.353	0.899
Sex	1.209	3.311	0.392 ~ 27.966	0.272
Hypertension	0.021	0.697	0.005 ~ 93.580	0.885
Coronary heart disease	0.078	0.369	0.015 ~ 9.366	0.546
Diabetes	4.244	1.881	0.022 ~ 157.757	0.780
BNP	0.078	1.000	0.999 ~ 1.001	0.780
CRP	0.006	1.002	0.945 ~ 1.063	0.936
Heart rate	0.277	1.003	0.992 ~ 1.105	0.599
PR	3.228	0.940	0.879 ~ 1.006	0.072
QRS	0.038	1.009	0.918 ~ 1.110	0.845
QTc	3.225	0.948	0.895 ~ 1.005	0.073
Tpe	10.023	1.600	1.191 ~ 2.141	0.002
Roxadustat treatment	5.328	7.560	1.357 ~ 42.121	0.021

## Discussion

CKD is an increasingly urgent public health problem that is expected to grow at an annual rate of 8% worldwide, with the fastest growth in developing countries [17]. Among dialysis patients, the incidence and mortality of CVD are 5–20 times higher than in the general population [18]. The incidence of CKD and its inextricably linked cardiovascular disease pose health service and health policy challenges globally. Therefore, it is urgent to develop new comprehensive approaches to screen patients with CKD who may develop CVD or even SCD. In the present study, we showed that serum Roxadustat treatment could reduce Tpe/QT ratio in PD patients.

Previous studies have demonstrated the prevention of CVD risk in CKD patients by lowering blood pressure and glucose [19, 20]. In recent years, some studies have begun to find new methods to predict the occurrence of CVD in patients with CKD and improve the prognosis of patients. Lees et al*.* showed a strong association between serum cystatin C (eGFRcys) and CVD and mortality, which can be used as part of cardiovascular risk assessment [21]. According to a review conducted by Wang et al., inflammation is a critical link between chronic kidney disease (CKD) and cardiovascular disease, playing a key role in the development of cardiovascular complications in CKD patients. Disrupting the vicious cycle of inflammation and disease could have a positive impact on patient prognosis [22]. Other studies have focused on the association of ECG indicators with patient prognosis. Among them, the ratio of QT interval to T peak to T end interval, Tpe/QT, reflects the imbalance between depolarization and repolarization of ventricular muscle and is one of the important indicators to assess the risk of ventricular arrhythmias [23, 24]. It has been shown that increased Tpe/QT values are associated with the development of ventricular arrhythmias, and cardiovascular events in dialysis patients are also strongly associated with ventricular arrhythmias [25]. Another study of dialysis patients found a positive correlation between increased Tpe/QT values and the incidence of cardiovascular events. Specifically, each 0.1 unit increase in Tpe/QT values was associated with an approximately 30% increase in the risk of cardiovascular events [26]. In addition, a study found that Tpe/QT values were significantly higher in dialysis patients than in the normal population, suggesting that dialysis patients have an imbalance between ventricular muscle depolarization and repolarization, which increases the risk of cardiovascular events [27]. These results all indicated the important role played by the Tpe/QT ratio in dialysis patients. So we recorded the Tpe/QT ratio in the ECG of PD patients in Roxadustat group and conventional group trying to find the effect of Roxadustat on Tpe/QT ratio.

Roxadustat can improve the dispersion of myocardial repolarization in dialysis patients, so it may improve myocardial fibrosis, myocardial damage and the incidence of malignant arrhythmias in dialysis patients. This may be related to Roxadustat inhibiting lyproline hydroxylase, thereby inhibiting the degradation of hypoxia-inducible factor HIF, improving angiogenesis, atherosclerosis, reducing oxidative stress and inflammation, thus protecting and stabilizing cardiomyocyte function. In addition, Roxadustat can act directly on ion channels in cardiomyocytes. Vascular endothelial cell (VEC) is directly in contact with blood. When the body is hypoxic, VEC can respond quickly to hypoxic stimulation. Hypoxia-induced vascular endothelial cell injury is an important factor in the pathophysiology of ischemic cardiomyopathy such as myocardial infarction and coronary atherosclerotic heart disease (CHD). Vascular endothelial growth factor (VEGF) can promote angiogenesis and induce the growth of vascular endothelial cells. [28]. Roxadustat upregulates VEGF/VEGFR2 through the HIF pathway and improves the tolerance of cardiomyocytes to ischemia and hypoxia by promoting vascular remodeling, the formation of collateral circulation and changing myocardial metabolism [29]. Our trial showed that PD patients who were treated with Roxadustat had a lower Tp-e/QT ratio. In addition, Roxadustat treatment was a risk factor for PD patients with a high Tp-e/QT ratio. This present research also has some limitations. First, this is a single-center study. Second, we included only a small study population. Thirdly, we have not studied the major cardiac outcomes.

## Conclusion

In summary, our results indicated that Roxadustat could improve ventricular repolarization in peritoneal dialysis patients, which indicated a potential cardiovascular protective effect of Roxadustat. Additionally, Tpe and Roxadustat treatment were the risk factors of PD patients with high Tp-e/QT ratio. This study might provide a new integrated approach to the assessment and treatment of CKD.

## Data Availability

All data can be requested from the authors.
